# Updates and advances in multiple sclerosis neurotherapeutics

**DOI:** 10.2217/nmt-2021-0058

**Published:** 2022-10-31

**Authors:** Moein Amin, Carrie M Hersh

**Affiliations:** 1 Cleveland Clinic, Department of Neurology, Cleveland, OH 44195, USA; 2 Cleveland Clinic, Lou Ruvo Center for Brain Health, Las Vegas, NV 89106, USA

**Keywords:** autologous hematopoietic stem cell transplantation, BTK inhibitors, disease-modifying therapy, early highly effective therapy, escalation therapy, multiple sclerosis, neuroprotective agents, progressive multiple sclerosis, relapsing multiple sclerosis, remyelinating therapies

## Abstract

The multiple sclerosis (MS) neurotherapeutic landscape is rapidly evolving. New disease-modifying therapies (DMTs) with improved efficacy and safety, in addition to an expanding pipeline of agents with novel mechanisms, provide more options for patients with MS. While treatment of MS neuroinflammation is well tailored in the existing DMT armamentarium, concerted efforts are currently underway for identifying neuropathological targets and drug discovery for progressive MS. There is also ongoing research to develop agents for remyelination and neuroprotection. Further insights are needed to guide DMT initiation and sequencing as well as to determine the role of autologous stem cell transplantation in relapsing and progressive MS. This review provides a summary of these updates.

## Plain language summary

The range of treatment options available for multiple sclerosis (MS) is growing, with the aim of developing safer and more effective therapies. There are ongoing efforts to discover additional mechanisms of MS and create drugs that can target these pathways. A more tailored approach will allow better personalization of drug selection for patients. There is currently a special focus on identifying treatment targets for progressive MS, where there are only a limited number of therapeutic options available to date. In addition, there is ongoing research aimed at developing stem cell therapies, drugs that provide neuroprotection and agents that can potentially reverse the damage caused by MS through remyelination. In this review, these topics are summarized.

**Practice points**

**Overview of available disease-modifying therapies (DMTs)**

- There have been major advancements and growth in the number of disease-modifying therapies with various efficacy and safety profiles for relapsing and progressive multiple sclerosis (MS).
- With the exception of teriflunomide, oral and infusion DMTs are considered more efficacious than platform self-injectable therapies.
- Use of highly effective therapies, such as monoclonal antibodies, must be balanced with potential safety risks.
- Newer generation sphingosine-1-phosphate receptor modulators have more selective receptor binding, resulting in reduced off-target effects.
- In the EXPAND clinical trial, siponimod demonstrated a reduction in confirmed disability progression at 3 and 6 months in patients with secondary progressive MS.
- Newer-generation fumarates, diroximel fumarate and monomethyl fumarate, have fewer gastrointestinal-related adverse events compared with dimethyl fumarate.
- Studies indicate that extended-interval dosing of natalizumab preserves comparable high efficacy to standard-interval dosing, while substantially decreasing the overall risk of progressive multifocal leukoencephalopathy.
- Ocrelizumab and ofatumumab are anti-CD20 monoclonal antibodies that are high-efficacy agents in the treatment of relapsing MS (RMS) and may have better safety than rituximab.

**Treating relapsing–remitting & progressive MS**

- ‘No evidence of disease activity’ is a commonly used ‘treat-to-target’ measure of disease freedom in relapsing forms of MS, and several variations in refining the parameters of this measure may make goals more stringent.
- Currently, there is clinical equipoise on two DMT initiation paradigms in early relapsing–remitting MS, which led to the development of two large pragmatic clinical trials to evaluate whether early highly effective therapy leads to better long-term outcomes versus a traditional escalation approach.
- While neuroaxonal degeneration is likely the major biological substrate of progression, discovery of chronic active inflammation, slowly expanding lesions and paramagnetic rim lesions has led to a more advanced understanding of the underlying pathophysiology of progressive disease, paving the way for new drug developments.

**Remyelinating strategies**

- Several therapeutic agents are currently being investigated for their potential role in remyelination and restoration of function.
- Two agents that initially showed promise – opicinumab (anti-LINGO-1) and high-dose biotin (MD1003) – did not meet pivotal clinical trial end points.

**Autologous hematopoietic stem cell transplantation**

- Autologous hematopoietic stem cell transplantation has gained interest in treating relapsing and progressive MS.
- Phase II studies showed promising results, especially in younger, active and treatment-refractory patients with no significant comorbidities and a phase III clinical trial evaluating autologous hematopoietic stem cell transplantation versus best available therapy (BEAT-MS) is ongoing.

**Emerging neurotherapeutics in the pipeline**

- A novel class of MS neurotherapeutics, BTK inhibitors, targets the adaptive and innate immune systems in the periphery and CNS via modulation of B lymphocytes and microglia.
- Currently, there are several ongoing phase III clinical trials investigating BTK inhibitors in both relapsing and progressive MS.
- Ublituximab, a glycoengineered anti-CD20 monoclonal antibody, demonstrated favorable disease freedom in phase II and phase III clinical trials, and its shorter infusion time than other B cell-depleting therapies may offer better convenience and access for patients.

## Introduction: multiple sclerosis pathophysiology, diagnosis & treatment

Multiple sclerosis (MS) is an immune-mediated, inflammatory, demyelinating and neurodegenerative disorder of the CNS that affects 1 million people in the USA and more than 2 million people worldwide [1,2]. The pathophysiology and etiology of MS is complex, with several environmental, infectious (e.g., Epstein–Barr virus), genetic, nutritional (e.g., vitamin D deficiency) and epigenetic components potentially playing a causative role in onset and disease course [3,4,5]. The inciting cause of MS inflammation is speculative, but is believed to be multifactorial, with genetic and environmental influences creating an adaptive T- and B-lymphocyte-mediated autoimmune response against the CNS [6]. The classic pathological description of MS lesions includes perivenular inflammatory demyelination consisting of T lymphocytes, B lymphocytes and plasma-cell infiltrates [7,8]. Progressive neurodegeneration can occur separately from acute inflammation and is characterized by axonal loss and white and gray matter atrophy [8]. While neuroaxonal degeneration is likely the major biological substrate of progression, there is increasing understanding that chronic inflammation also contributes to tissue loss and disability accumulation [9].

As the understanding of MS disease has evolved, so has the breadth of available treatments. To better reflect underlying MS pathology and corresponding clinical and radiographic activity, the definition of clinical phenotypes was recently updated [10,11]. Each MS subtype can further be categorized based on ‘activity’ and ‘progression’, with ‘activity’ defined as ongoing inflammation (e.g., new relapses, new gadolinium-enhancing [GdE] lesions and/or new/enlarging T2 lesions) and ‘progression’ determined via disability accumulation [12]. This is important because inflammatory activity and disability accumulation can occur in both relapsing and progressive MS and recognizing this balance can help guide treatment initiation and sequencing.

Newer disease-modifying therapies (DMTs) are available for treating MS, with various mechanisms of action, administration and dosing, and efficacy and adverse event (AE) profiles. DMTs are now approved for clinically isolated syndrome (CIS), relapsing–remitting MS (RRMS), active secondary progressive MS (SPMS) and primary progressive MS (PPMS). Treatment for progressive forms of MS without activity (e.g., without relapses and/or new MRI lesions) is more difficult, and therapeutic options are currently limited, resulting in one of the most significant unmet needs in the field. However, with further understanding of more distinct targets in the CNS that drive progressive disease, immunomodulatory therapies that simultaneously reduce acute and chronic neuroinflammation – Bruton’s tyrosine kinase (BTK) inhibitors – are actively being studied in later-phase clinical trials for both relapsing and progressive MS. In addition to preventing inflammatory and progressive disease, several strategies aim to target remyelination, repair and recovery and the MS field continues to make advances in autologous hematopoietic stem cell transplantation (AHSCT).

The rapidly growing number of available DMTs increases treatment options for patients with MS and represents an opportunity for personalized care. Concurrently, the growing body of literature and ongoing efforts in this space can be challenging to review and consolidate for real-world decision-making at the bedside. This review summarizes the evolution of neurotherapeutics in the current MS landscape and discusses the expanding treatment pipeline and future perspectives on contemporary DMT approaches. This will enable more informed decision-making regarding personalized selection of appropriate therapies for patients.

## Overview of available DMTs

The overall goal of DMTs is to reduce early clinical and subclinical disease activity that may contribute to long-term disability [13]. To date, there are 20 approved DMTs for MS in the USA. Overall, these agents have a wide array of neurological mechanisms that target distinct areas of the immune-mediated disease process. Tables 1–5 summarize the available DMTs approved in the USA.

### Immunomodulatory agents

#### IFN-β therapies & glatiramer acetate

In randomized, placebo-controlled trials, IFN-β and glatiramer acetate (GA) demonstrated similar reductions in annualized relapse rates (ARRs) by about 30%, decreased the severity of relapses and showed beneficial effects on MRI measures of disease activity [14]. While IFN-β1a demonstrated an effect against disability progression, this effect was not observed with IFN-β1b or GA [15,16]. Otherwise known as ‘platform’ therapies, which are summarized in Table 1 [14,16,17,18,19]; interferons and GA are moderately tolerated, and 20–30 years of accumulated data and clinical experience suggest strong long-term safety. Overall, the use of these therapies has decreased over time owing to the development of newer therapeutics with higher efficacy and better tolerability.

**Table 1.** Summary of drug administration, dosing, results of pivotal clinical trials, adverse events and safety monitoring for interferons and glatiramer acetate.

Disease-modifying therapy	Route and dose	Pivotal clinical trial results	Adverse events	Safety monitoring	Ref.
Glatiramer acetate	Subcutaneous: 20 mg once daily or 40 mg three times per week	Comparison: placebo ARR: ↓29% MRI disease activity: not adequately assessed Disability progression (EDSS): Not significant	Minor: injection-site reactions and postinjection vasodilator reaction Major: lipoatrophy, skin necrosis, anaphylaxis	No specific labs, skin surveillance	[17]
IFN-β1b	Subcutaneous: 0.25 mg every other day	Comparison: placebo ARR: ↓34% MRI disease activity: GdE lesions: ↓83% T2 lesions: ↓75% Disability progression (EDSS): ↓29% (insignificant change from baseline)	Minor: flu-like symptoms, headache, transaminitis, depression Major: suicidal ideation, anaphylaxis, hepatic injury, worsening rheumatological conditions, congestive heart failure, blood dyscrasias, seizures, autoimmune hepatitis	CBC with differential, LFTs, thyroid function tests, signs/symptoms of depression, skin surveillance	[18]
IFN-β1a	Subcutaneous: 22 or 44 μg three times weekly Intramuscular: 30 μg once weekly	Low dose 22 μg three times weekly Comparison: placebo ARR: ↓29% MRI disease activity: GdE lesions: ↓22% T2 lesions: ↓67% Disability progression (EDSS): ↓32% High dose 44 μg three times weekly Comparison: placebo ARR: ↓32% MRI disease activity: GdE lesions: ↓67% T2 lesions: ↓78% Disability progression (EDSS): ↓28% 30 μg once weekly Comparison: placebo ARR: ↓18% MRI disease activity: GdE lesions: ↓32% T2 lesions: ↓34% Disability progression (EDSS): ↓36%	Minor: flu-like symptoms, headache, transaminitis, depression, injection-site reactions Major: suicidal ideation, anaphylaxis, hepatic injury, worsening rheumatological conditions, congestive heart failure, blood dyscrasias, seizures, autoimmune hepatitis	CBC with differential, LFTs, thyroid function tests, signs/symptoms of depression, skin surveillance	[14,16]
PEGylated IFN-β1a	Subcutaneous: 125 μg every 14 days	Comparison: placebo ARR: ↓27% MRI disease activity: GdE lesions: ↓86% T2 lesions: ↓67% Disability progression (EDSS): ↓38%	Minor: flu-like symptoms, headache, transaminitis, depression, injection-site reactions Major: suicidal ideation, anaphylaxis, hepatic injury, worsening rheumatological conditions, congestive heart failure, blood dyscrasias, seizures, autoimmune hepatitis	CBC with differential, LFTs, thyroid function tests, signs/symptoms of depression, skin surveillance	[19]

ARR: Annualized relapse rate; CBC: Complete blood count; EDSS: Expanded Disability Status Scale; GdE: Gadolinium-enhancing; LFT: Liver function test.

#### Sphingosine-1-phosphate receptor modulators

Fingolimod was the first oral DMT approved for relapsing MS (RMS) in the USA in 2010. Fingolimod reduces circulating naive and central memory T and B lymphocytes by binding to sphingosine-1-phosphate (S1P) receptor and blocking lymphocyte egression from lymph nodes [20]. Sequestration of autoreactive lymphocytes prevents their recirculation to the CNS, thus inhibiting one of the primary steps in MS pathogenesis. In addition, it exerts anti-inflammatory and neuroprotective effects through interaction with astrocytes and S1P_5_ receptor on oligodendrocytes [21]. In phase III clinical trials, fingolimod showed a 54% reduction in ARR and slowed disability worsening by 30% versus placebo [22]. In a 1-year head-to-head clinical trial against intramuscular IFN-β1a, fingolimod demonstrated greater reduction in ARR by approximately 30% and reduction in MRI activity but no difference in disability worsening [23]. However, multiple off-target effects of fingolimod (e.g., S1P_3_, S1P_4_ and S1P_5_) may lead to unwanted AEs, including cardiac effects and macular edema. The favorable efficacy profile of fingolimod therefore led to an interest in developing small-molecule S1P receptor modulators with shorter half-life, preserved efficacy and greater S1P receptor selectivity which would reduce AEs.

Siponimod, a second-generation S1P receptor modulator, was approved for treatment of relapsing forms of MS (CIS, RRMS and active SPMS) in the USA in 2019 and is dosed based on the *CYP2C9* genotype. Unlike fingolimod, siponimod does not require activation via phosphorylation [24]. One of the major drawbacks of fingolimod is atrioventricular nodal blockage and bradycardia, mediated by S1P_3_ located on cardiac myocytes and the activation of inwardly rectifying G-protein-gated atrial potassium channel I [25], requiring first-dose observation when starting treatment. Siponimod, on the other hand, selectively binds to S1P_1_ and S1P_5_ [24]. Therefore, only patients with a cardiac history are recommended a first-dose observation when starting treatment. Further, in animal models, siponimod exerted independent neuroprotective effects, suggesting that it can target the neurodegenerative aspects of MS [26] and offering possible benefit in progressive disease. As such, the EXPAND clinical trial compared the efficacy of siponimod versus placebo in preventing disability progression in patients with SPMS [27]. In this study, patients receiving siponimod demonstrated a reduction in confirmed disability progression (CDP) at 3 months (hazard ratio [HR]: 0.79) and 6 months (HR: 0.74) with risk reductions of 21 and 26%, respectively [27]. This benefit was sustained as demonstrated in the EXPAND extension study [28]. Although S1P_5_ expressed by CNS cells, including oligodendrocytes, is a potential target in progressive MS, the benefit in EXPAND was greatest for younger patients with active disease. Based on these findings, siponimod was approved in the USA for active SPMS.

In addition to siponimod’s effects on the inflammatory and neurodegenerative components of MS, preclinical data also indicated that its neuroprotective mechanisms could include attenuation of axonal demyelination and promotion of axonal remyelination via oligodendrocytes [29]. Further, Jackson *et al.* demonstrated that after insult was elicited by lysophosphotidyl choline in a rat model, fingolimod could also actively promote remyelination, suggesting a direct interaction of fingolimod with microglia, oligodendrocytes and/or astrocytes [30]. In another model by the same group, siponimod, but not a selective S1P1 agonist, produced significantly greater myelin levels in demyelinated CNS components from rats versus a control group [30]. These data suggest that siponimod’s effects on remyelination could be mediated, at least in part, by S1P_5_ agonism.

Ozanimod, approved for RMS in the USA in 2020, is another second-generation selective S1P receptor modulator binding to S1P_1_ and S1P_5_ with high affinity [31]. Two large phase III trials, SUNBEAM and RADIANCE, compared the efficacy of ozanimod versus that of IFN-β1a in patients with RRMS [32,33]. In the RADIANCE trial, adjusted ARR was lower for ozanimod 1 mg daily (ARR: 0.17; 95% CI: 0.14–0.21) and ozanimod 0.5 mg daily (ARR: 0.22; 95% CI: 0.18–0.26) compared with IFN-β1a (ARR: 0.28; 95% CI: 0.23–0.32). More patients treated with IFN-β1a developed treatment-emergent AEs leading to discontinuation than patients treated with ozanimod [33]. Similar findings were reported in the SUNBEAM clinical trial [32], demonstrating that patients with RMS treated for at least 12 months with ozanimod outperformed patients treated with IFN-β1a via lower relapse rates. SUNBEAM also demonstrated significantly less cortical gray matter volume loss with ozanimod versus IFN-β1a (p < 0.0001). When evaluating matching adjusted indirect comparisons of safety and efficacy trial outcomes, ozanimod versus fingolimod was associated with a lower risk of conduction abnormalities (including atrioventricular block), mean lymphocyte count reduction, hepatotoxicity, 1-year risk of any AEs and 2-year risk of AEs leading to discontinuation [34].

To date, ponesimod is the latest highly selective S1P_1_ receptor modulator approved for RMS in the USA in 2021 [35]. The phase III clinical trial OPTIMUM showed that RRMS patients treated with ponesimod versus teriflunomide had lower ARR, with a relative rate reduction of 30.5%, lower rate of new GdE lesions and new/enlarging T2 lesions with a relative risk reduction of 56%, lower rate of brain volume loss by 0.34%, and reduced time to 12- and 24-week confirmed disability accumulation with risk estimates of 17 and 16%, respectively [36]. Further, ponesimod demonstrated favorable NEDA-3 (‘no evidence of disease activity’, defined as absence of relapses, MRI activity and disability progression) with an odds ratio of 1.70 (p < 0.001). Although AEs were similar across both treatment groups, discontinuation due to AEs was more common in ponesimod-treated versus teriflunomide-treated patients (8.7 vs 6.0%) [36].

Overall, fingolimod has the highest half-life (~6–9 days) when compared with other S1P receptor modulators and does not require titration [37]. In comparison, siponimod has a shorter half-life (around 30 h), and because its metabolism can be affected through the CYP2C9 enzyme, genotype testing and appropriate titration are required prior to initiation of this drug [38]. Ponesimod and ozanimod both have shorter half-lives (~33 and 21 h, respectively), and initiation of these agents also requires titration [39,40]. In addition to their shorter half-lives, selective S1P receptor modulators also exert more rapid lymphocyte recovery after withdrawal [41,42,43]. Ponesimod primarily exerts its effects through S1P_1_ receptor modulation compared with other available S1P receptor modulators. Although the reduction in bradycardia seen with siponimod, ponesimod and ozanimod versus fingolimod could partly be attributed to the enhanced selectivity of S1P receptor modulation, it could also be due to the presence of a titration schedule and their shorter half-lives (Table 2).

**Table 2.** Summary of drug administration, dosing, results of pivotal clinical trials, adverse events and safety monitoring for sphingosine-1-phosphate receptor modulators.

Disease-modifying therapy	Route and dose	Pivotal clinical trial results	Adverse events	Safety monitoring	Ref.
Fingolimod	Oral 0.5 mg once daily	Comparison: placebo ARR: ↓54% MRI disease activity: GdE lesions: ↓82% T2 lesions: ↓75% Disability progression (EDSS): ↓32% Comparison: intramuscular IFN-β1a ARR: ↓38% (1.25 mg) and 52% (0.5 mg) MRI disease activity: GdE lesions: ↓ mean 0.14 vs 0.51 (1.25 mg) and 0.23 vs 0.51 (0.5 mg) T2 lesions: ↓ mean 1.5 vs 2.6 (1.25 mg) and 1.7 vs 2.6 (0.5 mg) Disability progression (EDSS): not significant	Minor: lymphopenia (absolute lymphocyte count >200), transaminitis, infections, headache Major: bradycardia, heart block, hypertension, risk of infections (herpetic, PML, *Cryptococcus* *meningitis*), lymphopenia (absolute lymphocyte count <200), macular edema, skin cancer, reactive airway, PRES	First-dose cardiac monitoring, eye and skin examinations, CBC with differential, LFTs, VZV IgG prior to starting medication, PFTs (if clinically indicated), monitor BP during treatment	[22,23]
Siponimod	Oral Dose titration (for 2 mg maintenance, daily): 5-day titration, 0.25 mg days 1 and 2, 0.5 mg day 3, 0.75 mg day 4, 1.25 mg day 5 Dose titration (for 1 mg maintenance, daily): 4-day titration, 0.25 mg days 1 and 2, 0.5 mg day 3, 0.75 mg day 4 Maintenance dose: 1 or 2 mg once daily depending on *CYP2C9* genotype	Comparison: placebo ARR: ↓55% MRI disease activity: GdE lesions: ↓82% T2 lesions: ↓80% Disability progression (EDSS): ↓21% 3-month CDP (primary end point) ↓26% 6-month CDP	Minor: lymphopenia (absolute lymphocyte count >200), transaminitis, infections, headache Major: lymphopenia (absolute lymphocyte count <200), bradycardia and bradyarrhythmia at treatment initiation, macular edema, hypertension, VZV reactivation, PML	Initial dose titration mitigates cardiac first-dose effects (FDO per clinical judgment) *CYP2C9* genetic test, eye and skin examinations, CBC with differential, LFTs, VZV IgG prior to starting medication, PFTs (if clinically indicated), monitor BP during treatment	[27]
Ozanimod	Oral Dose titration (daily): 0.23 mg days 1–4, 0.46 mg days 5–7, 0.92 mg day 8 and thereafter Maintenance dose: 0.92 mg once daily	Comparison: IFN-β1a ARR: ↓48% and 38% MRI disease activity: GdE lesions: ↓63% and 53% T2 lesions: ↓48% and 42% Disability progression (EDSS): ↓10.8% 3-month CDP ↓8.6% 6-month CDP	Minor: lymphopenia (absolute lymphocyte count >200), transaminitis, infections (nasopharyngitis, URI), headache Major: lymphopenia (absolute lymphocyte count <200), bradycardia and bradyarrhythmia at treatment initiation, macular edema, hypertension, VZV reactivation, PML	Initial dose titration mitigates cardiac first-dose effects (FDO per clinical judgment) Eye and skin examinations, CBC with differential, LFTs, VZV IgG prior to starting medication, PFTs (if clinically indicated), monitor BP during treatment	[32,33]
Ponesimod	Oral Dose titration (daily): 2 mg days 1–2, 3 mg days 3–4, 4 mg days 5–6, 5 mg day 7, 6 mg day 8, 7 mg day 9, 8 mg day 10, 9 mg day 11, 10 mg days 12–14 Maintenance dose: 20 mg once daily day 15 and thereafter	Comparison: teriflunomide ARR: ↓31% MRI disease activity: combined unique active lesions: ↓56% Disability progression (EDSS): ↓17%	Minor: lymphopenia (absolute lymphocyte count >200), transaminitis, infections, headache Major: lymphopenia (absolute lymphocyte count <200), bradycardia and bradyarrhythmia at treatment initiation, macular edema, hypertension, VZV reactivation, PML	Initial dose titration mitigates cardiac first-dose effects (FDO per clinical judgment) Eye and skin examinations, CBC with differential, LFTs, VZV IgG prior to starting medication, PFTs (if clinically indicated), monitor BP during treatment	[36]

ARR: Annualized relapse rate; BP: Blood pressure; CBC: Complete blood count; CDP: Confirmed disability progression; EDSS: Expanded Disability Status Scale; FDO: First-dose observation; GdE: Gadolinium-enhancing; LFT: Liver function test; PFT: Pulmonary function test; PML: Progressive multifocal leukoencephalopathy; PRES: Posterior reversible encephalopathy syndrome; URI: Upper respiratory infection; VZV: Varicella zoster virus.

#### Teriflunomide

Teriflunomide was approved for RMS in the USA in 2012. It exerts its effects in MS via inhibition of *de novo* pyrimidine synthesis through selective and reversible inhibition of dihydroorotate dehydrogenase, yielding reduced proliferation of activated T and B lymphocytes [44]. Two randomized clinical trials, TEMSO and TOWER, demonstrated efficacy of teriflunomide versus placebo (Table 3) [45,46]. In the TENERE trial, there were no differences between teriflunomide and IFN-β1a 44 μg subcutaneous three times per week (SQ tiw) in time to first confirmed relapse nor time to treatment discontinuation due to any cause. The results also showed comparable ARR between teriflunomide 14 mg and IFN-β1a; however, ARR was significantly higher in the teriflunomide 7 mg group [47]. In the TOPIC clinical trial, patients with CIS randomized to either teriflunomide 14 mg (HR: 0.574; 95% CI: 0.379–0.869; p = 0.0087) or 7 mg (HR: 0.628; 95% CI: 0.416–0.949; p = 0.0271) had reduced risk of relapse that defined conversion to clinically definite MS, compared with placebo [48]. Common AEs include transaminitis, gastrointestinal (GI) disturbances (e.g., diarrhea, nausea and abdominal pain), hair thinning and headaches [45,46]. Hepatotoxicity is a potential risk, based on reports of liver failure in leflunomide [49]. Teriflunomide is considered teratogenic even if the patient is male; therefore, counseling regarding reliable contraception is warranted prior to treatment initiation. However, reassuring pregnancy observational data have been reported, including absence of spontaneous abortions and absence of increased prevalence of major malformations in teriflunomide-exposed newborns [50].

**Table 3.** Summary of drug administration, dosing, results of pivotal clinical trials, adverse events and safety monitoring for teriflunomide and cladribine.

Disease-modifying therapy	Route and dose	Pivotal clinical trial results	Adverse events	Safety monitoring	Ref.
Teriflunomide	Oral 7 or 14 mg once daily	7 mg once daily Comparison: placebo ARR: ↓31% MRI disease activity: GdE lesions: ↓57% T2 lesions: ↓44% Disability progression (EDSS): ↓20% (not significant) 14 mg once daily Comparison: placebo ARR: ↓32% MRI disease activity: GdE lesions: ↓80% T2 lesions: ↓77% Disability progression (EDSS): ↓26%	Minor: gastrointestinal side effects, nausea, hair thinning Major: transaminitis, teratogenicity (men and women), latent tuberculosis, neuropathy, hypertension	CBC with differential and LFTs (baseline and monthly for first 6 months) and then periodically, PPD or TB QuantiFERON prior to starting Rapid elimination available via cholestyramine or activated charcoal (if needed)	[45]
Cladribine	Oral Cumulative dose of 3.5 mg/kg divided into two treatment courses (1.75 mg/kg per treatment course); each treatment course is divided into two treatment cycles over 2 years	Comparison: Placebo ARR: ↓58% (3.5 mg/kg), ↓55% (5.25 mg/kg) MRI disease activity: GdE lesions: ↓86% T2 lesions: ↓73% Combined UAL: ↓74% Disability progression (EDSS): ↓47% 6-month CDP	Minor: URI, headache, fatigue Major: liver toxicity, hematological toxicity, lymphopenia, infections, risk of VZV reactivation, malignancy, risk of teratogenicity, graft-versus-host-disease	CBC with differential before first and second courses, 2 and 6 months after each course (absolute lymphocyte count >200 cells prior to second course), LFTs, VZV IgG prior to DMT, HIV, HBV and HCV panels, TB Quantiferon, skin check, baseline MRI (within 3 months of initial dose, rule out PML)	[51]

ARR: Annualized relapse rate; CBC: Complete blood count; CDP: Confirmed disability progression; EDSS: Expanded Disability Status Scale; GdE: Gadolinium-enhancing; HBV: Hepatitis B virus; HCV: Hepatitis C virus; LFT: Liver function test; PML: Progressive multifocal leukoencephalopathy; PPD: Purified protein derivative; TB: Tuberculosis; UAL: Unique active lesions; URI: Upper respiratory infection; VZV: Varicella zoster virus.

#### Fumarates

Dimethyl fumarate (DMF) was the third oral DMT approved for RMS in the USA in 2013. DMF is a fumaric acid ester that exerts its effects in MS by activating NRF2, an important transcription factor involved in maintaining redox homeostasis within cells [52]. In addition, fumarates reduce neuronal excitotoxicity, modulate transendothelial migration across the blood–brain barrier and exert an impact on the peripheral immune system [53]. DMF demonstrated clinical efficacy in phase III trials, with a 48–53% reduction in ARR as compared with placebo [54,55]. The most frequent AEs observed in clinical trials were GI symptoms – generally more prominent during the first several weeks of treatment and mitigated by taking DMF with food – and transient skin flushing mitigated by aspirin [56]. As compared with clinical trials, DMF discontinuation in real-world observational studies was more prevalent. For example, one study demonstrated that at 24 months, 25% of DMF-treated patients discontinued drug, versus 12–16% of DMF-treated patients in phase III clinical trials [57]. Another observational study showed that GI side effects were observed in approximately 40% of DMF-treated patients, and approximately 9% of patients discontinued therapy due to AEs [58].

Because of concerns regarding DMF-associated AEs, two newer fumarates with similar dosing frequency were recently approved in the USA: diroximel fumarate (DRF; 2019) and monomethyl fumarate (MMF, 2020). Comparable to DMF, DRF exhibits anti-inflammatory effects through activating NRF2 but has a different chemical structure and higher molecular weight. In this context, it is thought to improve tolerability by reducing GI AEs [59,60]. DRF undergoes esterase cleavage to MMF, the same pharmacologically active metabolite as DMF. Given similar bioavailability to DMF, no direct comparative effectiveness trials were conducted prior to approval, but EVOLVE-MS-1 is an ongoing phase III, open label, 96-week, single-arm study evaluating the efficacy, safety and tolerability of DRF in RRMS [61]. Interim efficacy data showed a low adjusted ARR (0.16 at 48 weeks) compared with baseline (95% CI: 0.13–0.20) and a reduction in mean GdE lesions by 77% at 48 weeks (p < 0.0001) [61]. In patients receiving DRF, GI AEs typically occurred within the first month of treatment, were mild to moderate in severity, lasted about 7.5 days for most patients and only led to discontinuation in 0.7% of patients [53]. EVOLVE-MS-2 was a phase III, randomized, double-blind, head-to-head study comparing GI tolerability of DRF versus that of DMF in RRMS [60]. The results of this study showed that in DRF-treated versus DMF-treated patients, GI tolerability was improved, with less severe AEs, fewer days of self-reported symptoms and lower rates of drug discontinuation due to intolerability [60]. MMF also demonstrated improved GI tolerability compared with DMF in a randomized, double-blind, head-to-head clinical trial [62]. In this context, DRF and MMF may be reasonable options for patients who have adequate disease control on a fumarate but have intolerable DMF-associated GI AEs.

**Table 4.** Summary of drug administration, dosing, results of pivotal clinical trials, adverse events and safety monitoring for fumarates.

Disease-modifying therapy	Route and dose	Pivotal clinical trial results	Adverse events	Safety monitoring	Ref.
Dimethyl fumarate	Oral Titration dose: 120 mg twice daily × 7 days Maintenance dose: 240 mg twice daily	Comparison: placebo ARR: ↓44–53% MRI disease activity: GdE lesions: ↓74–90% T2 lesions: ↓71–85% Disability progression (EDSS): ↓38%	Minor: flushing, gastrointestinal symptoms Major: transaminitis, lymphopenia, PML	Baseline and every 6 months: CBC with differential, LFTs	[54,55]
Diroximel fumarate	Oral Titration dose: 231 mg twice daily × 7 days Maintenance dose: 462 mg twice daily	Interim data available Adjusted ARR: 0.16 GdE lesions: ↓77%	Minor: flushing, lower risk of gastrointestinal symptoms compared with DMF Major: transaminitis, lymphopenia, PML (presumed risk as DMF); similar to DMF	Baseline and every 6 months: CBC with differential, LFTs	[60]
Monomethyl fumarate	Oral Titration dose: 95 mg twice daily × 7 days Maintenance dose: 190 mg twice daily	–	Minor: flushing, lower risk of gastrointestinal symptoms compared with DMF Major: transaminitis, lymphopenia, PML (presumed risk as DMF); similar to DMF	Baseline and every 6 months: CBC with differential, LFTs	

ARR: Annualized relapse rate; CBC: Complete blood count; DMF: Dimethyl fumarate; EDSS: Expanded Disability Status Scale; GdE: Gadolinium-enhancing; LFT: Liver function test; PML: Progressive multifocal leukoencephalopathy.

#### Monoclonal antibodies

Monoclonal antibodies in MS are considered highly effective therapies. This group of treatments has the advantage of being administered less frequently than oral (e.g., S1PR modulators, teriflunomide and fumarates) and platform self-injectable agents. However, their higher efficacy needs to be balanced against greater safety risks [63]. This warrants consideration of the drugs’ risk and benefit profiles in addition to the patient’s demographics, baseline disease characteristics and personal preferences in order to develop an individualized treatment plan. Traditionally, monoclonal antibodies were more commonly used in RMS that had not responded to first-line treatments and/or patients who demonstrated highly active disease with elevated risk of early progression [64]. However, improvements in safety profiles and risk mitigation strategies of approved monoclonal antibody therapies make their first-line use increasingly common.

**Table 5.** Summary of drug administration, dosing, results of pivotal clinical trials, adverse events and safety monitoring for monoclonal antibody therapies.

Disease-modifying therapy	Route and dose	Pivotal clinical trial results	Adverse events	Safety monitoring	Ref.
Natalizumab	Intravenous 300 mg every 4 weeks	Comparison: Placebo ARR: ↓68% MRI disease activity: GdE lesions: ↓92% T2 lesions: ↓83% Disability progression (EDSS): ↓42%	Minor: Infusion-related reactions, headaches, joint pain, fatigue, wearing-off phenomenon Major: PML, infections (URI, UTI), Herpes Zoster, liver failure	CBC with differential, LFTs, serum JCV antibody (every 6 months; every 3 months if JCV antibody positive), annual MRI brain, natalizumab antibodies (if clinically warranted)	[65]
Alemtuzumab	Intravenous First course: 12 mg IV daily × 5 days Second course: 12 mg IV daily × 3 days, 12 months from first course Third course (as needed): 12 mg IV daily × 3 days when appropriate per disease course	Comparison: IFN-β1a ARR: ↓55% and 49% MRI disease activity: GdE lesions: ↓63% and 61% T2 lesions: ↓17% and 32% Disability progression (EDSS): ↓27% (non-significant) and 42%	Minor: infusion-related reactions Major: secondary autoimmune disease (thyroid dysfunction, ITP, Goodpasture syndrome, hepatitis), lymphopenia, infections (HSV, VZV, Listeria), acute coronary syndrome, hemophagocytic lymphohistiocytosis, hemorrhagic and ischemic strokes, malignancy, PML	Monthly CBC with differential, LFTs, urinalysis with microscopy, TSH and free T4 every 3 months, SCr for 48 months after final infusion (REMS Program) Also recommended: annual skin exam, annual HPV screening and gynecological exam	[66,67]
Ocrelizumab	Intravenous Initial dose: 300 mg IV followed by 300 mg IV 2 weeks after initial dose Maintenance dose: 600 mg IV every 6 months	Comparison: IFN-β1a ARR: ↓46% and 47% MRI disease activity: GdE lesions: ↓94% and 95% T2 lesions: ↓77% and 83% Disability progression EDSS: Pooled result: ↓40%	Minor: infusion-related reactions, mild infections (URI/UTI) Major: severe infusion-related reactions, reactivation of HBV, severe or recurrent infections, malignancy	HBV panel, CBC with differential, LFTs prior to starting treatment; CBC and CMP periodically; immunoglobulin levels at baseline and periodically	[68]
Ofatumumab	Subcutaneous Initial dosing: 20 mg administered at weeks 0, 1 and 2 Subsequent dosing: 20 mg administered monthly, starting at week 4	Comparison: Teriflunomide ARR: ↓51% and 58% MRI disease activity: GdE lesions: ↓97% and 94% T2 lesions: ↓82% and 85% Disability progression (EDSS): ↓34%	Minor: injection-related reactions, local injection-site reactions, reduction in immunoglobulins, mild infections (upper respiratory tract), headaches Major: recurrent or serious infections, HBV reactivation	CBC with differential, LFTs, HBV panel and quantitative serum immunoglobulin screening before first dose; regular monitoring during treatment	[69]

ARR: Annualized relapse rate; CBC: Complete blood count; CMP: Complete metabolic profile; EDSS: Expanded Disability Status Scale; GdE: Gadolinium-enhancing; HBV: Hepatitis B virus; HPV: Human papillomavirus; HSV: Herpes simplex virus; ITP: Immune thrombocytopenia; IV: Intravenous; JCV: John Cunningham virus; LFT: Liver function test; PML: Progressive multifocal leukoencephalopathy; REMS: Risk Evaluation Mitigation Strategy; SCr: Serum creatinine; TSH: Thyroid-stimulating hormone; URI: Upper respiratory infection; UTI: Urinary tract infection; VZV: Varicella zoster virus.

#### Natalizumab

Natalizumab, the first approved infusion therapy for RMS in the USA (approved in 2004), works through inhibition of α4 integrins and prevents leukocyte migration across the blood–brain barrier [70]. By blocking VLA-4, natalizumab allows fewer inflammatory cells to enter the brain and thereby blunts CNS inflammation typical of MS. Results from phase III clinical trials demonstrated that natalizumab reduces clinical relapses by 67%, new brain MRI lesions by 83% and risk of CDP by 42% [65,71]. These results have been further validated in real-world, long-term observational studies [72,73,74,75,76]. Natalizumab is a generally well-tolerated agent. Common side effects include infusion-related reactions, headache, fatigue, GI side effects and upper respiratory and urinary tract infections. Given its mechanism of action, natalizumab use is primarily limited due to the higher risk of progressive multifocal leukoencephalopathy (PML) in patients previously exposed to John Cunningham virus (JCV) [77,78].

PML is a progressive brain infection caused by the reactivation of JCV. The risk of PML in natalizumab-treated patients was initially reported to be 1/1000 in 2006 [79]. However, PML risk can be stratified using clinical data. The risk of natalizumab-associated PML is increased in patients with positive JCV antibody, duration of therapy greater than 24 months and prior use of immunosuppressive therapy [79,80]. Accordingly, the risk is highest in patients with all three risk factors, estimated at 11.1 PML cases per 1000 patients, and lowest in those with negative JCV antibody serostatus (≤0.09 PML cases per 1000 patients) [79]. In the absence of a positive antibody titer, there is no evidence that greater treatment duration is associated with increased PML risk. In addition to the above three factors, JCV antibody index is another measure that can assist in risk stratification. For example, patients diagnosed with PML tend to have higher indices than patients without PML [81]. Overall, among natalizumab-treated patients with seropositive JCV antibody status and no prior immunosuppressive use, the lowest risk is among those with index ≤0.9 and on treatment for 1–24 months (0.1 PML cases per 1000 patients), and the highest risk is among patients with index >1.5 and on treatment >48 months (5.4 PML cases per 1000 patients) [81].

In summary, the JCV antibody index may assist with risk stratification, though various factors are also involved in determining natalizumab use and safety on an individualized basis. Interestingly, a recent study indicated that patients significantly overestimated the risk of natalizumab-related PML (0.1–87%; mean 31.5%) compared with actual risk (0.01%) based on published risk estimates in accordance with JCV antibody index, duration of time on therapy and prior use of immunosuppressive agents [82]. These data suggest that patients would benefit from accurate PML risk education by the MS provider to aid in the shared decision-making process when considering natalizumab.

A group of neurologists hypothesized that by reducing the amount of natalizumab administered, perhaps there could be some desaturation of lymphocytes, allowing a sufficiently small amount of retrafficking to keep JCV out of the CNS. To answer this question, a study was conducted using retrospective analysis of the TOUCH^®^ database – a restricted distribution program for monitoring safety in patients treated with natalizumab in the USA – to determine whether extended-interval dosing (EID) versus standard-interval dosing (SID) every 4 weeks was associated with lower risk of PML. The results demonstrated that overall risk was significantly reduced, by approximately 90%. Overall PML incidence for EID treatment >48 months was ∼1.5/1000 patients versus ∼4/1000 patients on SID treatment [83]. Further, the 2-year phase IIIb NOVA study showed that 6-week EID provided a high level of radiographic efficacy in controlling disease activity in patients who switched from SID after at least 12 months without relapses under the primary estimand (mean numbers of new or enlarging T2 lesions at 72 weeks was 0.2 in EID vs 0.05 in the SID group; mean lesion ratio: 4.24; p = 0.076) [84]. Using the secondary estimand, the numerical difference in new/enlarging T2 lesions reached statistical significance. Of note, interpretation of any statistical difference is limited because disease activity in the SID group was lower than expected. Another study investigated a 12-week extended dosing interval to investigate whether CD4 lymphocyte counts correlated with different phases of natalizumab treatment. Investigators observed significant shifts in CD4 counts, whereby lymphocytes were increased from baseline while patients were on treatment and returned back to baseline off treatment, only to return again upon natalizumab reinitiation. Further, the 12-week natalizumab interruption was reassuringly well tolerated and not associated with disease recrudescence or disability progression [85].

Importantly, natalizumab withdrawal is associated with a significant risk of disease recrudescence, which can occur despite initiation of an alternative DMT [86]. This risk can be reduced by switching to another high-efficacy DMT, preferably within 2 months of natalizumab cessation [87]. It is noteworthy that the risk of disease reactivation postnatalizumab is quite variable and depends on a number of factors, including the mechanism of action of the next DMT and the time interval off natalizumab. A meta-analysis indicated that younger age, higher number of relapses and GdE lesions prior to natalizumab start, and fewer natalizumab infusions were correlated with increased risk of natalizumab-withdrawal reactivation [86].

### Continuous lymphocyte-depleting agents

#### Anti-CD20 monoclonal antibody therapies

Although MS was traditionally considered a T lymphocyte-mediated disorder, there is substantial evidence of the distinct role of B lymphocytes in MS pathophysiology, leading to the development and use of targeted B cell-depleting therapies. Rituximab, a chimeric monoclonal antibody targeting CD20 (which is primarily expressed on B lymphocyte surfaces and a minority of T lymphocytes), has been used off-label to treat patients with MS [88]. Anti-CD20 monoclonal antibodies exert their beneficial effects in MS through rapid and effective elimination of circulating B lymphocytes [89]. Despite rituximab’s widespread use in MS, no large phase III trials have examined its effects in relapsing forms of MS, but this area is unlikely to be pursued in the setting of multiple newly developed anti-CD20 therapies.

##### Ocrelizumab

Ocrelizumab – a recombinant, humanized anti-CD20 monoclonal antibody that binds to a different but overlapping epitope compared with rituximab – was approved in the USA in 2017 for treatment of MS [90]. Developed after the observation that rituximab was effective in MS, ocrelizumab demonstrated effectiveness in both RRMS and PPMS. Clinical efficacy of ocrelizumab in RRMS was demonstrated in two identical randomized trials, OPERA I and OPERA II [68,91]. These studies showed that compared with IFN-β1a, ocrelizumab was associated with significantly reduced ARR (by 45%) and reduced risk of CDP (by 34%) at 6 months; it significantly reduced the number of GdE lesions (by 95%) and new/enlarging T2 lesions (by about 80%) [68]. In addition to its benefits in RRMS, ocrelizumab was the first DMT approved for PPMS based on the results of ORATORIO [92,93]. In this placebo-controlled clinical trial, ocrelizumab demonstrated reduction in CDP by 24%. Secondary outcome measures – including reduction in timed 25-foot walk, volume of T2-weighted lesions, number of new/enlarging T2 lesions and brain volume loss – were also favorable [92]. It is noteworthy that ocrelizumab was evaluated in a younger patient population (≤55 years of age), of whom 27.5% had GdE lesions at baseline.

Combined safety analysis of OPERA I, OPERA II, ORATORIO and extension and real-world studies demonstrated that ocrelizumab is generally well tolerated [91,93]. The most common side effects include infusion-related reactions (34–40%) – which are mitigated by pretreatment with antihistamines, acetaminophen and methylprednisolone prior to each infusion – and mild infections (e.g., upper respiratory and urinary tract infections and nasopharyngitis) which respond well to antibiotics [91,93]. Compared with rituximab, ocrelizumab has the potential for decreased infusion-related reactions due to its humanized origin.

Serious infections can also occur (two cases per 100 patient-years) and mostly consist of urinary tract infections/urosepsis, pneumonia or cellulitis [94]. In an analysis of patients treated with ocrelizumab for up to 7 years in the pivotal clinical trials or in real-world post-marketing settings, overall rates of AEs and serious AEs were comparable to those reported for placebo and IFN-β1a [94]. It is important to note that patients in the real world versus restricted trial settings tend to be older, have longer disease duration, have a greater number of prior DMTs, make up a higher proportion of progressive MS and have more comorbidities. In this context, similar low reporting of serious infections among ocrelizumab-treated versus IFN-β1a- and placebo-treated patients across multiple trial and real-world settings is reassuring for relatively safe use in clinical practice. There were also low rates of ocrelizumab discontinuation due to AEs, suggesting favorable long-term tolerability [94].

There is a complex interplay between the differential immunological effects of ocrelizumab across the B- and T-cell lines that may increase the overall risk of infections but is not currently well understood. There is an apparent association between decreased IgG levels and rates of serious infections, but the type, severity, duration and outcomes of these infections are similar to those in the overall ocrelizumab and general MS populations [94]. Other risk factors such as age, body mass index, other comorbidities, prior DMTs and disability may also be influential [89]. Overall, the significance of hypogammaglobulinemia and long-term use of anti-CD20 therapies for long-term safety risks is not well understood and deserves further exploration.

To date, there have been 12 cases of PML reported in ocrelizumab-treated patients: nine were previously on natalizumab, one was previously on fingolimod, and there have been two non-carryover cases of ocrelizumab-related PML [95]. There is also an increased risk of herpes-related infections (5%), for which treatment during an active infection is contraindicated. Hepatitis B reactivation can occur; thus, viral screening prior to DMT initiation is warranted. Based on findings from clinical trials, there was a small increased risk of malignancy (<1%), specifically breast cancer in women, though trial extension and real-world data suggest comparable risk with the MS population and non-significant increased risk compared to the general population. Routine age-appropriate malignancy screening is recommended [94].

##### Ofatumumab

Ofatumumab was the first fully human anti-CD20 monoclonal antibody to be approved for use in MS, in 2020. Efficacy of ofatumumab in RRMS patients was demonstrated in two identical phase III, double-blind, randomized clinical trials (ASCLEPIOS I and ASCLEPIOS II) which were conducted in parallel [69]. These studies demonstrated that, compared with oral teriflunomide, ofatumumab was associated with relative reduction of ARR by approximately 55%, relative reduction of GdE lesions by about 95%, relative reduction of new/enlarging T2 lesions by approximately 83% and, in a pooled analysis, reduction in 3- and 6-month confirmed disability worsening by about 33%. However, there was no difference in slope of brain volume change from baseline between treatments [69]. Injection-related reactions, particularly with the first one, were more frequent with ofatumumab (20.2%) versus the dummy injection with teriflunomide (15.0%), requiring premedication with the first injection in less than 70% of patients. Serious infections were rare in the respective groups (2.5 vs 1.8%). Real-world safety experience requires ongoing monitoring.

### Time-limited immunosuppression followed by immune repopulation

#### Alemtuzumab

Alemtuzumab, approved for RMS in the USA in 2014, is a humanized monoclonal antibody directed against CD52. Alemtuzumab induces rapid depletion of CD52+ B and T lymphocytes with subsequent repopulation of cells comprising a distinct lymphocyte repertoire, including memory B and T lymphocytes and regulatory T lymphocytes [96]. Phase III clinical trials CARE-MS I and CARE-MS II demonstrated high relapse-free rates (78 and 65%, respectively), compared with subcutaneous IFN-β1a, with a significant reduction in the number of new T2 and GdE lesions and reduction in brain volume loss in previously treated MS, including patients with breakthrough disease and disability (CARE-MS II) [66,67]. An extension study of pooled CARE-MS I and CARE-MS II patients with highly active disease on alemtuzumab confirmed sustained efficacy at 9-year follow-up, including 62% with NEDA, 69% with freedom of MRI activity, 50% with 6-month confirmed disability improvement and a median cumulative change in brain volume of -2.15% [97].

However, the risk of significant safety events mandates the need for strict monitoring through patient enrollment in the Risk Evaluation Mitigation Strategy program. Patients undergo monthly monitoring via blood and urine testing to monitor for secondary autoimmune conditions – such as thyroid disease (34% of patients in clinical studies), immune thrombocytopenic purpura (2% of patients) and glomerular basement membrane disease (0.3% of patients) – during and after the course of therapy [98]. Some other major AEs include thyroid cancer (0.3% of patients), opportunistic infections, and ischemic and hemorrhagic stroke (possibly mediated through blood pressure elevation). Age-appropriate malignancy screening, including annual gynecological and skin evaluations, is also a key component of risk monitoring. Due to the risk of herpes infections, antiviral prophylactic treatment with acyclovir 200–400 mg twice daily is recommended during the course of alemtuzumab until recovery of CD4 lymphocytes to >200 cells/μl, with a minimum treatment duration of 2 months even if CD4 lymphopenia resolves earlier [99]. Patients are also instructed to avoid potential sources of *Listeria monocytogenes* owing to a case that emerged in an open-label clinical trial, attributed to eating unpasteurized cheese [100].

Several retrospective real-world studies evaluated the safety and efficacy of alemtuzumab, demonstrating profiles similar to those of phase III trials [101,102,103,104]. When compared with cladribine, although alemtuzumab had similar efficacy, it was associated with higher rates of AEs [105]. Further, a propensity score-adjusted study evaluated alemtuzumab performance versus IFN-β, fingolimod and natalizumab in RRMS patients treated up to 5 years in the MSBase registry. Overall, alemtuzumab showed superior efficacy in ARR versus IFN-β or fingolimod but was similar to natalizumab in mitigating relapse activity. The probability of disability improvement was similar across alemtuzumab, IFN-β and fingolimod. However, natalizumab appeared superior to alemtuzumab in allowing disability recovery [106].

#### Cladribine

Oral cladribine was approved for RMS in the USA in 2019. It is a deoxyadenosine analog that disrupts DNA metabolism and causes apoptosis, preferentially depleting peripheral B and T lymphocytes due to their high levels of enzyme necessary for incorporation into DNA [107,108]. Efficacy and safety of cladribine in RMS were investigated in two randomized controlled trials, CLARITY and ORACLE MS [51,109]. In CLARITY, cumulative doses of 3.5 and 5.25 mg/kg were evaluated, demonstrating 57.6% and 54.5% reductions in ARR versus placebo, respectively. Further, patients treated with cladribine demonstrated higher relapse-free rates (79.7% for 3.5 mg/kg and 78.9% for 5.25 mg/kg) compared with placebo (60.9%), relative reduction in risk of 3-month disability progression by 33% (3.5 mg/kg) and 31% (5.25 mg/kg), and concomitant reductions in brain atrophy rate, number of GdE lesions and active T2 lesions (Table 3) [51]. Importantly, a 2-year extension study demonstrated that the reduction in ARR was durable even after cladribine discontinuation [110]. In ORACLE MS, cladribine was compared with placebo in patients with CIS [109]. Patients treated with cladribine demonstrated a significant reduction in time to conversion to clinically definite MS (67% for 3.5 mg/kg and 62% for 5.25 mg/kg). Cladribine 3.5 mg/kg was also assessed in a 96-week phase II study, ONWARD, as add-on therapy for patients still experiencing active RMS despite IFN-β treatment. The results showed that cladribine/IFN-β reduced relapses and MRI lesion activity versus placebo/IFN-β but was associated with an increased incidence of lymphopenia [111].

### Summary of DMT use in clinical practice

Overall, oral DMTs are considered more efficacious than platform self-injectable therapies, aside from teriflunomide, which demonstrated similar efficacy to IFN-β1a [47]. Safety concerns for oral therapies are increased compared with self-injectable platform agents, including the rare risk of PML with S1P receptor modulators and fumarates (∼1/18,000 with fingolimod and 1/50,000 with DMF) [112] and the rare risk of melanoma and cutaneous lymphoma with fingolimod, hepatotoxicity, lymphopenia and infections [22,23,27,32,33,36,45,51,54,55,60.

Natalizumab is a reasonable choice in patients with active disease and is increasingly used as first-line therapy in seronegative JCV antibody cases. In JCV antibody-positive patients, the risks and benefits need to be carefully weighed in an individualized manner. Utilizing EID may substantially reduce the overall risk of PML.

Anti-CD20 monoclonal antibody therapies are reasonable treatment options for first-line use, given their favorable safety profiles to date (especially in those with highly active disease), and second-line use in RMS patients who have demonstrated breakthrough disease, especially in the setting of positive JCV antibody serology, in whom other highly effective treatments (e.g., natalizumab) may carry higher risks. However, in the appropriate clinical context, initiating natalizumab in JCV antibody-positive patients can be considered if the appropriate risk mitigation strategies are employed. In PPMS, ocrelizumab is the only approved DMT, though its efficacy in patients older than 55 years and those without evidence of active inflammatory disease remains unclear.

## Treating relapsing–remitting MS

Pathogenesis and disability accumulation in MS are a result of both neuroinflammation and neurodegeneration. With increasing understanding of disease pathophysiology and the discovery of new DMTs, there is an ongoing need to determine the optimal goals for treatment and measuring outcomes. In the subset of patients who undergo AHSCT, disease-free-survival has been used as one of the main measures of treatment success. It is defined as the time until patient death or evidence of disease activity (e.g., CDP, clinical relapse or new MRI lesions) [113].

However, when evaluating DMTs, NEDA has become a commonly used ‘treat to target’ approach. This concept emerged following a *post hoc* analysis of the AFFIRM trial of natalizumab and was originally referred to as ‘disease activity-free status’ [114]. This measure later evolved to NEDA-3, representing the absence of relapses, MRI activity and progression of disability as measured by the expanded disability status scale (EDSS) [115,116,117]. However, there are now several variations in refining the parameters of NEDA [118]. As NEDA-3 is more suited to measure the neuroinflammatory aspects of MS, additional measures that better incorporate components of neurodegeneration and cognitive disability have been proposed [119]. NEDA-4, which includes brain atrophy as a fourth dimension, was thus introduced, as an additional element of neurodegeneration that may better capture disease progression [120]. Incorporating serum neurofilament light chain as a fifth dimension for including a biomarker of disease activity has also been raised for the purpose of defining NEDA [118]. Further dimensions may appear, such as patient-reported outcomes and MRI lesion morphology, as more nuanced measurements of disease activity and progression are incorporated [121].

However, given that MS DMTs are primarily focused on reducing inflammation, NEDA is a treatment target that is somewhat difficult to achieve in clinical practice and only becomes more stringent with added dimensions. This may lead to excessive rates of ‘treatment failures’ and DMT switching. In contrast, one must consider that without stringent markers such as NEDA, one may miss the ‘window of treatment opportunity’ if a DMT is not appropriately switched/escalated, potentially leading to irreversible disability accumulation [122]. Switching therapies after disability accrual is suboptimal. When breakthrough disease occurs (e.g., relapse, new T2/GdE lesions and/or disease progression), it is prudent to consider a DMT with a different mechanism of action that may improve therapeutic response. Currently, there are no biomarkers available to determine which DMT class would be most suitable at the individual level. Instead, best clinical judgment based on the patient’s demographics, disease characteristics and comorbidities; risk stratification; accessibility and personal preferences are vital when selecting a DMT.

Despite our improved understanding of measuring disease outcomes, determining the optimal strategy to achieve freedom from disease has been challenging due to the heterogeneity and complexity of MS pathophysiology. This challenge is also superimposed on the rapidly growing number of safe and effective neurotherapeutics for MS, with 20 DMTs available to date. Currently, there is no formal consensus on the fundamental principles of initial treatment selection in early MS, nor on DMT sequencing approaches. As such, there is variable use of DMTs in clinical practice. Further, the complexity of treatment choices can seem overly daunting, which can lead to therapeutic inertia. In this context, there is clearly a growing need for personalized and evidence-based treatment approaches.

Currently, there are two common therapeutic paradigms in treating MS: escalation approach (initial medication with lower efficacy and a lower chance of serious AEs, subsequently switching to a more effective medication in the setting of breakthrough disease) and early highly effective treatment approach (initial medication of higher efficacy but with an increased chance of serious AEs). Presently, there is clinical equipoise on whether early highly effective therapy leads to better long-term outcomes versus traditional escalation. To answer this vital question, two large multicenter, pragmatic clinical trials were designed and are currently ongoing: DELIVER-MS (NCT03535298) [123] and TREAT-MS (NCT03500328). The results of these studies will help guide overall treatment philosophy and have important implications for personalized DMT choices.

As stated above, there are no formal guidelines on sequencing DMTs. Switching therapies should be approached in a shared decision-making manner between the clinician, patient and care partner, taking into consideration a variety of factors, including goals of treatment, disease activity, prior DMT history and concomitant medications, and personal preferences. Reasons to discuss DMT switch include: breakthrough disease activity/inadequate response to therapy, intolerability/side effects, AEs, pregnancy planning, nonadherence, psychosocial reasons and economic/financial barriers. In the event of breakthrough disease on compliant DMT, a more potent agent with higher efficacy and different mechanism of action should be considered rather than agents with similar or lower efficacy, to reduce risk of relapse occurrence [124].

## Treating progressive MS & neuroprotective agents

The predominant pathological hallmark of RRMS is a breakdown of the blood–brain barrier that allows infiltration of activated lymphocytes, resulting in focal CNS inflammatory demyelination [7,8]. On the other hand, progressive MS is primarily driven by neurodegeneration of the white and gray matter that yields brain and spinal cord atrophy, superimposed on mild-to-moderate inflammation [8]. Factors that contribute to neurodegeneration include: mitochondrial dysfunction that yields impairments in the oxidative phosphorylation pathway, causing chronic hypoxia [125]; iron accumulation over time in myelin and oligodendrocytes, causing oxidative injury [126] and chronic radial expansion of microglia and macrophages at lesion edges (i.e., chronic active or slowly expanding lesions) [127]. In addition, both B and T lymphocytes likely play a part in driving progressive MS (particularly meningeal and cortical inflammation) leading to neuronal loss and can potentially represent a therapeutic target [128].

Although clinical relapses are a hallmark of RRMS, some evidence indicates that disability accumulation may occur independently of relapse activity. This concept challenges the phenotypic distinction of relapsing and progressive MS as two discrete entities [129]. The concept of progression independent of relapse activity (PIRA) was first introduced in 2018, with PIRA defined as worsening disability independent of relapses within a defined period or in relapse-free patients [130]. Given the inherent challenges of understanding disease pathophysiology and identifying effective CNS targets, lack of validated outcome measures of disease progression and the mostly negative clinical trial experiences to date, there has been relatively little success in treating progressive MS, specifically PIRA. This is considered one of the most significant unmet needs for patients with MS [64].

There are ongoing global efforts in the development of neurotherapeutics for progressive MS through research supported by organizations such as the National Multiple Sclerosis Society, the International Progressive MS Alliance, the European Committee for Treatment and Research in Multiple Sclerosis and the Multiple Sclerosis Outcomes Assessment Consortium. In 2013 the definition of progressive disease was updated to better define disease activity and progression [11]. Early trials in MS were mostly negative, likely owing to the use of older immunosuppressive medications and variability in selection of participants and clinical outcomes. There is also the fundamental question of whether there are innate differences in the inflammatory response of relapsing versus progressive disease, given the failure of most anti-inflammatory therapies in progressive MS to date [125]. Several of these negative studies included agents such as intravenous immunoglobulin, fingolimod, GA, natalizumab, rituximab, IFN-β, cladribine, sulfasalazine, cyclophosphamide and azathioprine [131,132,133,134,135,136,137,138,139,140,141,142]. Two recent positive trials evaluated siponimod and ocrelizumab in progressive MS. In EXPAND, siponimod versus placebo reduced CDP in a well-represented population of patients with SPMS [27]. In ORATORIO, ocrelizumab reduced CDP in PPMS versus placebo [27,92].

Several agents have been proposed as possible neuroprotective agents. Compared with placebo, α-lipoic acid demonstrated a 68% reduction in annualized percentage change in brain volume in patients with SPMS [143], while high-dose simvastatin showed a 43% reduction in the annualized rate of whole-brain atrophy in the MS-STAT clinical trial [144]. More recently, ibudilast, a cyclic nucleotide phosphodiesterase inhibitor, was evaluated in patients with PPMS and SPMS in the SPRINT-MS trial [145]. The results showed that ibudilast versus placebo demonstrated lower rates of brain atrophy, measured as the rate of change in brain parenchymal fraction (p = 0.04), representing approximately 2.5 ml less brain tissue loss over a period of 96 weeks [145]. In the MS-SMART phase II trial, the effects of riluzole, amiloride and fluoxetine against placebo in patients with SPMS were evaluated, but the results were negative for all therapies [146].

## Remyelinating strategies

In addition to the expansion of immunotherapies to prevent acute tissue injury, another major goal of therapeutics development in MS is to repair damage and restore function. Many research efforts have focused on the remyelination of existing lesions, both to restore function and to prevent axonal degeneration [147]. Oligodendrocyte precursor cells (OPCs), which are widely distributed throughout the CNS, mediate myelination and remyelination after being recruited to areas of myelin injury and differentiate into myelinating oligodendrocytes [148]. While OPCs are abundant in the CNS and present in chronic MS lesions, remyelination is insufficient, especially in late-stage and progressive MS [149]. This insufficiency is likely driven by multiple factors, including mitochondrial dysfunction leading to excitotoxicity and a mismatched state of increased energy demand and reduced axonal ATP production, resulting in a chronic state of virtual hypoxia in chronically demyelinated axons [125]. In this context, age and inhibitory/toxic components of the lesion microenvironment appear to impair OPC differentiation, challenging neurotherapeutics development.

Typically, remyelination in clinical trials is measured through neuroimaging markers such as myelin water fraction, diffusion tensor imaging, magnetization transfer ratio (MTR) and PET; neurophysiological markers such as evoked potentials and other techniques such as optical coherence tomography [148]. There have been several clinical trials evaluating both clinical and radiological remyelination outcomes in MS and optic neuritis. While some have been negative [150,151] and many are ongoing, several notable results are discussed below and summarized in Supplementary Table 1 [150,152–165].

Research attention has focused on possible remyelinating agents that have the potential to stimulate OPC differentiation, such as antihistamines. In a prospective, open-label, single-blinded study of patients with RRMS and SPMS, the volume of tissue undergoing increase in voxel-wise MTR was significantly higher in natalizumab-treated compared with IFN-β1a-treated patients and healthy control subjects [166]. In the phase II trial ReBUILD – a randomized, placebo-controlled, crossover study – RRMS patients with chronic optic neuropathy were treated with clemastine fumarate, a first-generation antihistamine. These patients demonstrated reduced visual-evoked potential latency by 1.7 ms/eye (95% CI: 0.5–2.9; p = 0.0048), possibly through off-target antimuscarinic action [157]. In another phase II placebo-controlled trial, RRMS patients treated with GSK239512, an H3-receptor antagonist, demonstrated improvement in mean change in postlesion MTR in GdE lesions, possibly through histamine regulation of oligodendrocyte differentiation [158]. Though clinical meaningfulness has yet to be established from these clinical trials, they were important in introducing new treatment targets and promising methods for detecting lesion remyelination in RRMS.

Opicinumab, a human monoclonal antibody, also gained interest as a potential remyelinating agent through its antagonism of LINGO1, a negative regulator of oligodendrocyte differentiation. In the phase II, randomized, double-blind, placebo-controlled clinical trial RENEW, opicinumab-treated patients with a first episode of acute optic neuritis demonstrated an improvement of 7.6 ms in visual-evoked potential latency [159]. However, the subsequent phase II trial SYNERGY failed to demonstrate improvements in disability outcomes [160].

Remyelination in progressive MS is an attractive concept because of its theoretical ability not only to halt disability accumulation but also to potentially reverse and restore existing disability accrual. Biotin, a cofactor for essential carboxylases, was thought to support myelin repair by enhancing fatty acid synthesis and protecting hypoxia-driven axonal degeneration through augmentation of neuronal energy production. In this context, high-dose biotin (MD1003) was deemed to be a promising remyelinating agent. Results of the first pilot study in MD1003-treated patients with chronic progressive MS [167] and a subsequent randomized, double-blind, placebo-controlled trial (MS-SPI) were positive, demonstrating higher proportion of progressive patients with MS with sustained disability reversal [161]. However, in a definitive phase III clinical trial (SPI2), MD1003 did not demonstrate significant improvement in disability outcomes and is therefore no longer recommended for treatment of progressive MS [162].

## Autologous hematopoietic stem cell transplantation

In recent years, AHSCT has gained increasing interest in the treatment of MS. AHSCT aims to renew and regenerate the immune system through removal of autoreactive lymphocytes following high-potency immunosuppression to produce chemical immunoablation. The immune system is then repopulated using hematopoietic stem cells from the patient [168]. Due to potential serious AEs associated with immunoablation, AHSCT has primarily been reserved for patients with highly aggressive MS who have previously failed multiple DMTs. Many of the trials to date have been small, open-label studies with various immunoablative and transplant protocols and different treatment outcomes (Supplementary Table 1). However, several of these single-arm clinical trials and two phase II studies (NCT00273364, EUDRACT 2007-000064-24) offered supportive evidence to proceed with larger randomized-controlled trials comparing AHSCT to other aggressive DMTs [154,155]. In HALT-MS, the efficacy of AHSCT was evaluated in patients with MS with disability progression on DMT. The trial results demonstrated that AHSCT achieved event-free survival of 69.2%, progression-free survival of 91.3%, clinical relapse-free survival of 86.9% and MRI activity-free survival of 86.3% at 5 years [163].

Results from prior AHSCT trials demonstrated high relapse-free rates, positive MRI outcomes and stabilization or improvement of disability. These trials suggest AHSCT is most effective in patients with treatment-refractory, active RMS and in younger, ambulatory patients with relatively short disease duration and accruing disability progression [169]. Although the results are promising, it is noteworthy that these open-label trials employed careful patient selection and generally excluded patients with significant comorbidities. In a meta-analysis, pooled treatment-related mortality was 2.1% [170]. Based on prior studies, factors associated with higher mortality include progressive disease and higher EDSS scores. Encouragingly, AHSCT-associated mortality appears to have decreased over time, likely driven by greater experience and better patient selection [64]. As such, further phase III studies may indicate AHSCT as a viable strategy in aggressive MS. BEAT-MS (NCT04047628) is an ongoing, randomized trial evaluating the efficacy, safety and cost–effectiveness of AHSCT compared with other highly effective DMTs (e.g., natalizumab, anti-CD20 monoclonal antibodies and alemtuzumab). Such trials are needed to determine the optimal timing and role of AHSCT in treating MS within the rapidly growing neurotherapeutic landscape. These studies are also warranted given the lack of controlled clinical trial data to date and the potential financial gain of different ‘stem cell’ businesses benefiting from the experimental status for their liability protection.

## Emerging DMTs in the pipeline

As the understanding of MS pathophysiology continues to evolve, new research efforts are focused on immune pathways involving cells compartmentalized in the CNS. Chronic neuroinflammation and neurodegeneration contribute to accelerated brain volume loss and long-term disability accumulation across MS phenotypes, which is considered one of the most important unmet needs for patients with MS. B lymphocytes residing in the CNS of patients with MS release immune and inflammatory mediators. Microglia are CNS-resident cells of the innate immune system and play a crucial role in neuronal tissue damage and neuroinflammation in MS; activated microglia are involved in neurodegeneration and are associated with chronic active lesions, paramagnetic rim lesions, slowly expanding lesions and the chronic smoldering disease component of MS [9], which all play important roles in disease progression.

BTK is an essential enzyme in the activation pathways of microglia, macrophages and B lymphocytes, both in the periphery and in the CNS [171,172]. BTK inhibition in the CNS has the potential to directly modulate (rather than deplete) the activity of microglia, macrophages and B-lymphocytes, which could potentially address chronic inflammation and neurodegenerative processes. Moreover, by targeting B lymphocytes and myeloid cells both outside and inside the CNS, there may be greater and perhaps synergistic effects on inflammatory demyelination than with existing DMTs [171,172]. BTK inhibitors that are currently being investigated in MS phase III clinical trials include evobrutinib, tolebrutinib and fenebrutinib, each with distinct pharmacological profiles that differ in potency, selectivity and CNS distribution (Supplementary Table 1) [173].

Evobrutinib was evaluated in a randomized, double-blind, placebo-controlled phase II trial in patients with RMS. The results showed a lower total number of GdE lesions during weeks 12–24, with baseline-adjusted rate ratio 0.30 for evobrutinib 75 mg once daily (95% CI: 0.14–0.63). However, no changes in ARR or disability progression were detected with any evobrutinib dose [164]. Another phase IIb, randomized, double-blind, placebo-controlled trial evaluated the efficacy of tolebrutinib in RMS (defined as either relapsing–remitting or relapsing SPMS) and found a dose-dependent (85%) reduction in the number of new GdE lesions after 12 weeks of treatment [156]. A couple of important exploratory end points – reduction in the number of paramagnetic rim lesions and volume of slowly expanding/evolving lesions – were also of interest, based on the hypothesis that a CNS-penetrant immunomodulator might reduce otherwise treatment-resistant lesions associated with neuroinflammation and disability accumulation. However, no significant differences were observed over 12 weeks of tolebrutinib use [156], suggesting that longer-term data are necessary to capture treatment effects. There are currently many ongoing phase III clinical trials evaluating fenebrutinib in RMS (NCT04586023) and PPMS (NCT04544449); tolebrutinib (SAR442168) in RMS (NCT04410978 and NCT04410991), non-relapsing SPMS (NCT04411641) and PPMS (NCT04458051) and evobrutinib in RMS (NCT04338022 and NCT04338061).

In addition to identifying and treating novel targets for MS, there are ongoing efforts in improving the specificity and tolerability of available strategies. Ublituximab, a novel glycoengineered anti-CD20 monoclonal antibody that is currently being reviewed by the US FDA, demonstrated favorable outcomes in patients with RMS in a phase II placebo-controlled trial. Between 24 and 48 weeks, the results showed 93% relapse-freedom, no GdE lesions (p = 0.003) and 74% of patients with NEDA [165]. The ULTIMATE I and II phase III trials demonstrated superior efficacy in ublituximab- versus teriflunomide-treated patients with RMS via lower annualized relapse rates and fewer MRI lesions over 96 weeks but did not result in a significantly lower risk of disability [174]. The faster infusion time (1 h) and increased antigen-dependent cellular cytotoxicity associated with ublituximab versus complement-mediated cytotoxicity may result in improved convenience, access and tolerability compared with other anti-CD20 therapies.

## Conclusion

In recent years, many new and exciting advances in MS, including various provocative neurotherapeutics and drug targets, have been uncovered. Historically, MS treatment was largely concentrated on reducing the inflammatory features of the disease. While immunomodulatory therapies can result in considerable reductions in inflammation (e.g., relapse rates, new MRI lesions), these findings are offset by less impressive disability outcomes. With improved understanding of additional MS pathophysiological pathways, including the inception of PIRA, there are new goals and benchmarks for disease therapy. Currently, there is a large treatment gap for non-active progressive MS, and this is considered one of the most significant unmet needs in the field. As such, concerted efforts are focused on targeting neurodegenerative pathology with high-throughput screening for drug discovery for patients with progressive MS. Given that the existing DMTs primarily affect peripheral adaptive immunity, a new approach that combines peripheral and CNS immunomodulation is attractive. BTK inhibitors specifically are a provocative class of new therapies being investigated in MS that link therapeutic targets across the adaptive (e.g., B lymphocytes) and innate (e.g., myeloid cells) immune systems. As is currently being studied in late-phase clinical trials, targeting B lymphocytes and CNS-resident microglia may lead to clinically meaningful effectiveness for patients with both relapsing and progressive MS.

Other advances in the MS field continue to drive forward-thinking concepts. Incorporating more stringent goals for DMT response in RMS via NEDA has been recommended [118]; though the adoption of these goals may further challenge what is considered an appropriate therapeutic response. Others have proposed that ‘minimal evidence of disease activity’ may be a more realistic target [175]. Further works are needed to bridge and synchronize these gaps in heterogeneity of treatment effects and what are useful, meaningful and feasible drug therapy goals. There are also several ongoing trials evaluating the efficacy and safety of AHSCT for treatment of MS, particularly for patients with aggressive disease. Likewise, recent identification of several novel cellular targets modulating remyelination has propelled a research focus on the development of drugs promoting repair and neuroprotection.

The delicate balancing of MS disease and patient heterogeneity, recognizing clinical and paraclinical markers in prognosticating a deeply phenotyped cohort, and the wide availability of existing and emerging neurotherapeutics – all while balancing the impact of the current COVID-19 landscape – certainly challenges treatment. However, more importantly, these concepts provide a construct for evidence-generation solutions that ultimately allow continued advancements in our understanding of how to navigate these challenges for optimal personalized medicine. The field of MS is clearly rapidly expanding across a plethora of opportunities, which ultimately will lead to better individualized and accessible care for patients.

## Future perspective

Although the long-term safety profiles for platform injectable DMTs are well established and proven, there are concerns regarding the long-term use of more efficacious DMTs, such as risks of serious infections and malignancy. Further, the direct and indirect costs associated with long-term DMT use are significant, and some governmental payers may no longer cover DMTs after a certain age. In this context, DMT discontinuation may be considered, especially in older patients in whom the benefits may no longer outweigh the potential risks, or in those with prolonged inactive disease. However, many questions pose challenges in deciding when DMT discontinuation may be appropriate. How old should a patient be? Is chronological age more or less important than the presence of comorbidities that increase DMT risks? How much stability is ‘true stability’? How is stability measured?

To date, there is limited evidence on DMT discontinuation, though there is a growing body of literature to explore this strategy. In one observational study, time to relapse was similar in patients with >5 years of relapse-freedom on self-injectable treatment who discontinued DMT compared with a propensity-matched cohort of patients who continued DMT, although time to CDP was shorter [176]. In another retrospective observational study, most patients over the age of 60 years who discontinued DMT continued to remain off treatment, with only one clinical relapse observed in 178/600 patients who discontinued DMT [177]. However, due to inherent biases with real-world retrospective studies that limit interpretation, there is currently an ongoing randomized controlled trial (DISCOMS NCT03073603) evaluating DMT discontinuation compared with standard of care in patients >55 years old without evidence of disease activity, defined as no new clinical relapses for at least 5 years and no new MRI lesions for at least 3 years. This pivotal trial will provide valuable information on whether DMT discontinuation can safely be explored in a stable older patient population.
