# Supplementary material for: Updates and advances in multiple sclerosis neurotherapeutics
Source: Neurodegener Dis Manag. 2022 Oct 31;13(1):47–70. doi: 10.2217/nmt-2021-0058 (PMC10072078; doi:10.2217/nmt-2021-0058)
Supplement: Supplementary file 1 [file nmt-13-47-s1.docx]

Supplemental Table 1. Summary of possible and failed emerging multiple sclerosis therapies with relevant clinical trial details and outcomes.

| Agent/Study Name if Applicable | Clinical Trial Structure | Study Duration | Population | Summary of Outcomes |
| --- | --- | --- | --- | --- |
| **Remyelinating Agents** | | | | |
| GSK239512  (anti-histamine) [145] | Phase 2, randomized, double-blind, parallel-group, placebo-controlled | 48 weeks | RRMS | GSK239512 was associated with positive effect sizes of 0.344 and 0.243 for adjusted mean changes in the normalized MTR for GdE and Delta-MTR lesions, respectively. |
| Bexarotene  (retinoid X agonist) [139] | Phase 2,  randomized, double-blind, parallel-group, placebo-controlled | 6 months | RRMS | Change in mean lesional MTR was not different between bexarotene and placebo. All bexarotene-treated participants had at least one AE. |
| MD1003  (high dose biotin) [148]  MS-SPI | Randomized, double-blind, placebo-controlled | 12 months | SPMS or PPMS | Statistically higher proportion of patients in the MD1003 cohort achieved disability reversal. |
| MD1003  (high dose biotin) [137] | Randomized, double-blind, placebo-controlled | 6 months | RRMS or progressive MS | MD1003 did not significantly improve visual acuity. |
| MD1003  (high dose biotin) [149]  SPI2 | Phase 3 randomized, double-blind, parallel-group, placebo-controlled | 15 months | SPMS or PPMS | MD1003 did not significantly improve disability or walking speed in patients with progressive MS. |
| Clemastine  (anti-histamine) [144]  ReBUILD | Phase 2, randomized, double-blind, placebo-controlled, cross-over | 150 days | RMS | Clemastine fumarate reduced P100 latency delay by 1.7 ms/eye (p=0.0048). |
| Opicinumab  (anti-LINGO-1) [146]  RENEW | Phase 2, randomized, double-blind, placebo-controlled | 24 weeks | First unilateral ON | Change in VEP P100 latency in affected eye, referenced to the unaffected eye, did not differ significantly between opicinumab and placebo in ITT analysis. Significant improvement in latency was seen in PP analysis. |
| Opicinumab  (anti-LINGO-1) [147]  SYNERGY | Phase 2, randomized, double-blind, placebo-controlled, dose-ranging | 72 weeks | RMS | There was no significant dose-linear improvement in disability compared with placebo. |
| Domperidone  (dopamine antagonist) [140] | Phase 2 randomized, open-label, single-blind | 12 months | SPMS | Domperidone treatment could not reject futility in reducing disability progression. |
| **Autologous Hematopoietic Stem Cell Transplantation** | | | | |
| NCT00273364 [141] | Phase 2, randomized, nonmyeloablative AHSCT vs. continued DMT | 60 months | RRMS | Nonmyeloablative AHSCT compared with DMT resulted in a significantly prolonged time to disease progression. |
| EUDRACT No. 2007-000064-24 [142] | Phase 2, randomized,  AHSCT vs. mitoxantrone | 4 years | RRMS or SPMS | AHSCT compared with mitoxantrone reduced the number of new T2 lesions, GdE lesions, and ARR. |
| HALT-MS [150] | Phase 2,  single-arm, AHSCT | 5 years | RRMS | AHSCT demonstrated 91.3% progression-free survival, 86.9% relapse-free survival, and 86.3% MRI activity-free survival. Overall, AHSCT was effective for inducing long-term sustained remission of active RRMS at 5 years. |
| NCT04047628 (BEAT-MS) | Phase 3, randomized, controlled, AHSCT vs. best available therapy | 72 months | RRMS and SPMS | Ongoing  Primary Outcome: Relapse-Free Survival |
| **Monoclonal Antibodies** | | | | |
| Ublituximab  (anti-CD-20 monoclonal antibody) [152] | Phase 2, randomized,  placebo-controlled | 48 weeks | RRMS | Ublituximab demonstrated 93% relapse-freedom, no GdE lesions, and 74% with NEDA. |
| **Bruton’s Tyrosine Kinase (BTK) Inhibitors** | | | | |
| Evobrutinib [151] | Phase 2,  randomized,  double-blind,  placebo vs. evobrutinib vs. DMF | 24 weeks | RRMS | There were fewer GdE lesions with evobrutinib compared to placebo. |
| Tolebrutinib [143] | Phase 2,  randomized, double-blind,  placebo-controlled, cross-over, dose-finding | 16 weeks  (12 weeks on tolebrutinib) | RRMS | There was a dose-dependent reduction in new GdE lesions with tolebrutinib. |
| Fenebrutinib (NCT04586023)  FENhance 1 and 2 | Phase 3,  randomized,  double-blind, double-dummy, fenebrutinib vs. teriflunomide | 96 weeks | RMS | Ongoing  Primary Outcomes: ARR (up to 96 weeks), Time to 12-Week CDP |
| Fenebrutinib (NCT04544449)  FENtrepid | Phase 3,  randomized,  double-blind,  fenebrutinib  vs. ocrelizumab | 120 weeks | PPMS | Ongoing  Primary Outcome: Time to 12-Week CDP |
| Tolebrutinib  (NCT04410978 and NCT04410991)  GEMINI 1 and 2 | Phase 3,  randomized,  double-blind,  tolebrutinib  vs. teriflunomide | 18-36 months  (event-driven trial) | RMS | Ongoing  Primary Outcome: ARR up to 36 months |
| Tolebrutinib (NCT04411641)  HERCULES | Phase 3,  randomized,  double-blind,  placebo-controlled | 24-48 months  (event-driven trial) | NRSPMS | Ongoing  Primary Outcome: 6-Month CDP |
| Tolebrutinib (NCT04458051)  PERSEUS | Phase 3,  randomized,  double-blind,  placebo-controlled | 24-48 months  (event-driven trial) | PPMS | Ongoing  Primary Outcome: 6-Month CDP |
| Evobrutinib  (NCT04338022 and NCT04338061)  EVOLUTION 1 and 2 | Phase 3,  randomized,  double-blind,  evobrutinib  vs. teriflunomide | 96 weeks | RRMS | Ongoing  Primary Outcome: ARR |

Abbreviations: AE: adverse event; AHSCT: autologous hematopoietic stem cell transplantation; ARR: annualized relapse rate; CDP: confirmed disability progression; DMF: dimethyl fumarate; DMT: disease modifying therapy; GdE: gadolinium contrast enhancing; ITT: intention to treat; MTR: magnetization transfer ratio; NEDA: no evidence of disease activity; NRSPMS: non-relapsing secondary progressive multiple sclerosis; ON: optic neuritis; PP: per protocol; PPMS: primary progressive multiple sclerosis; RMS: relapsing MS; RRMS: relapsing-remitting multiple sclerosis; PMS: secondary progressive multiple sclerosis; VEP: visual evoked potential.
